# Influence of galantamine in the inflammatory process and tissular lesions caused by *Trypanosoma cruzi* QM2 strain

**DOI:** 10.1590/0037-8682-0201-2021

**Published:** 2021-11-12

**Authors:** Lucas Fadel Camargo, Guilherme Donzalisky Pinheiro, Priscilla Bianca de Oliveira, Daniele Moraes Losada, Eduardo Federighi Baisi Chagas, Márcia Aparecida Sperança, Agnaldo Bruno Chies, Maria Angélica Spadella, Luciamáre Perinetti Alves Martins

**Affiliations:** 1 Faculdade de Medicina de Marília, Curso de Medicina, Marília, SP, Brasil.; 2 Faculdade de Medicina de Marília, Departamento de Farmacologia, Marília, SP, Brasil.; 3 Universidade Estadual de Campinas, Departamento de Anatomia Patológica, Campinas, SP, Brasil.; 4 Faculdade de Medicina de Marília, Grupo de Estudo em Envelhecimento e Obesidade, Marília, SP, Brasil.; 5 Universidade Federal do ABC, Centro de Ciências Naturais e Humanas, São Bernardo do Campo, SP, Brasil.; 6 Faculdade de Medicina de Marília, Laboratório de Embriologia Humana, Marília, SP, Brasil.; 7 Faculdade de Medicina de Marília, Departamento de Parasitologia, Marília, SP, Brasil.

**Keywords:** Trypanosoma cruzi, Acethylcholinesterase, Butyrylcholinesterase, Inflammatory process, Colon, Chagas disease

## Abstract

**INTRODUCTION:**

*Trypanosoma cruzi* infection triggers an inflammatory process with exacerbated production of cytokines that stimulate inflammatory and anti-inflammatory signals, including the efferent anti-inflammatory signal known as the anti-inflammatory cholinergic pathway. Thus, the use of anticholinesterase drugs, such as galantamine, could minimize the inflammatory process caused by this disease.

**METHODS:**

For the study at 30, 60, and 90 days, 120 Swiss mice were divided into three groups. Each group was subdivided into four subgroups: uninfected/untreated (CTRL), uninfected/treated (GAL), infected/untreated (INF), and infected/treated (GAL/INF). The infected groups were inoculated intraperitoneally with 0.1 ml of mouse blood containing 5 × 10^4^ trypomastigote forms of the *T. cruzi* QM2 strain. The galantamine-treated groups received 5 mg/kg of galantamine orally, through pipetting. From each subgroup, the parameters of parasitemia, histopathological analysis, butyrylcholinesterase activity (BuChE), and functional study of the colon were evaluated.

**RESULTS:**

BuChE performance was observed when AChE was suppressed, with increased activity in the GAL/INF group similar to the INF group on the 30^th^ day post infection, thus corroborating the absence of a significant difference in parasitic curves and histopathological analysis.

**CONCLUSIONS:**

The presence of an inflammatory process and nests of amastigotes, as well as evidence of reactivity to ACh and NOR, suggest that galantamine did not interfere with the colonic inflammatory response or even in colonic tissue parasitism at this stage of Chagas disease.

## INTRODUCTION

Discovered more than 100 years ago, Chagas disease, caused by the protozoan *Trypanosoma cruzi*, affects approximately 6 to 7 million people from regions in Latin America, such as Argentina, Brazil, and Mexico, where it is considered endemic^1^. 

The clinical evolution of Chagas disease can be divided into two phases: acute and chronic. The acute phase begins shortly after infection and is characterized by fever, subcutaneous edema, lymphadenomegaly, hepatomegaly, and splenomegaly, among other manifestations, and may be oligosymptomatic in some situations^2^. After the acute phase, patients evolve to the chronic phase, which may be asymptomatic, without clinical manifestations and positive serology, called the indeterminate form^3^ ^-^ ^6^. Approximately 30% of the patients, however, develop the symptomatic chronic form, presenting with cardiac, digestive, or mixed manifestations, which are influenced by several factors such as the parasite inoculation route, host immune response, and tissue tropism of different strains of the parasite^2^ ^,^ ^7^ ^-^ ^9^. 

The cardiac and digestive forms of chronic Chagas disease are characterized by the enlargement of affected organs, known as megaviscerae, because of an inflammatory process triggered by the host's immune response, whose control has been explored in different ways^10^. Previous studies^11^ ^-^ ^13^ have shown that macrophages, among other cytokine-producing cells, have receptors for acetylcholine (ACh). The binding of ACh to its receptors triggers the inhibition of the inflammatory process, decreasing the release of pro-inflammatory cytokines such as TNF-α and interleukins 1, 6, and 8 (IL-1, IL-6, and IL- 8). In addition, some studies have shown that ACh acts on the vascular endothelium, decreasing adhesion and subsequent leukocyte trafficking to the inflammation site^13^ ^-^ ^14^. Thus, the use of anticholinesterase drugs could contribute to minimizing the inflammatory process and consequently, the clinical manifestations of Chagas disease by maintaining the ACh concentration at higher levels and reducing the inflammatory response in areas of vagal innervation^13^ ^-^ ^16^. In this context, ACh binds to the α7 subunits of the nicotinic receptors of cytokine-producing cells (α7nAChR), inhibiting the transcription of nuclear factor kB (NF-kB)^17^. The neural pathway for inflammation control is faster than the humoral pathway and is more sensitive, requiring a lower concentration of cytokines for an effective response^14^.

Galantamine is an alkaloid, selective, and competitive inhibitor of AChE that interacts allosterically with nicotinic acetylcholine receptors. This interaction mechanism potentiates the action of ACh receptor agonists^18^. Treatment of mice with induced endotoxemia^12^ and obesity^19^ with the use of 4 mg/kg of galantamine showed a reduction in IL-6 and TNF-α and a decrease in inflammation. The anti-inflammatory effect of galantamine was also observed in an experimental model of induced arthritis in rats, at a concentration of 5 mg/kg^20^. Similar results were found in a study conducted by Chies et al.,^21^ who showed a reduction in liver inflammatory damage after the induction of ischemia and reperfusion in rat livers with previous administration of rivastigmine. Infection of mice with the *T. cruzi* QM2 strain triggers a chronic inflammatory reaction in the colonic myenteric plexus with a discrete mural inflammatory process^22^. In this study, using the experimental model of Chagas infection in mice with the *T. cruzi* QM2 strain, the anti-inflammatory effect of the anticholinesterase-drug galantamine was investigated. 

## METHODS

### Animals

A total of 120 Swiss male mice with an average weight of 30 g and approximately 30 days of age from the Central Vivarium of the Faculty of Medicine of Marília (Famema), were used. The animals remained in the maintenance laboratory of the Famema Parasitology Discipline in an environment with controlled temperature (23-25 °C), light-dark cycle of 12/12 h, and water and food provided *ad libitum*. This study was approved by the Animal Research Ethics Committee of the Faculty of Medicine of Marília (protocol number 112/17). 

### Study design and animal experimental groups

In this study, the *T. cruzi* QM2 strain was used, which has shown high parasitemia and virulence in Swiss mice and a parasitic peak around the 25^th^ day after infection^22^. Thus, on the day of infection, treatment with galantamine was started at a dose of 0.5 mg/kg of weight, totaling 0.15 mg for each animal belonging to the groups treated throughout the experimental period. The first period studied was the 30^th^ day post infection (dpi) and the beginning of treatment, considered as the middle of the course of the disease evolution in its acute phase. The second period studied occurred after 60 dpi or the end of the acute phase and the third at 90 dpi, considered the beginning of the chronic phase. For this, 120 mice were randomly divided into three groups of 40 animals, which were analyzed after 30, 60, and 90 days of the experimental procedure. Subsequently, each group was subdivided into four subgroups: uninfected/untreated (CTRL: CTRL-30, CTRL-60, CTRL-90), uninfected/treated (GAL: GAL-30, GAL-60, GAL-90), infected/untreated (INF: INF-30-; INF-60; INF-90), and infected/treated (GAL/INF: GAL/INF-30; GAL/INF-60; GAL/INF-90).

### Infection and galantamine treatment protocols

Sixty mice belonging to the INF and GAL/INF groups were infected intraperitoneally with 0.1 ml of blood containing 5 × 10^4^ blood trypomastigote forms of the *T. cruzi* QM2 strain from another previously infected mouse. After infection, mice from all experimental groups were evaluated at 30, 60, and 90 days.

Mice from the GAL and GAL/INF groups were treated daily with galantamine at a concentration of 5 mg/kg of body weight^20^. The drug was diluted in mineral water and immediately administered to each animal orally by pipetting 10 μl of the solution.

### Parasitemia assessment

Parasitemia assessment begun on the 7^th^ dpi and was performed weekly, using the method of Brener^23^, during the acute phase of infection in five mice from the INF and GAL/INF groups, totaling approximately 10 counts for each animal.

### Sample collection

At 30, 60, and 90 dpi and treatment, the animals in each experimental group were euthanized in a CO_2_ chamber. One milliliter of blood was collected via cardiac puncture using a heparinized syringe, placed in a 1 ml Eppendorf® tube, centrifuged at 1300 g per minute for plasma separation, which was transferred to another tube and frozen at -80 °C. Simultaneously, two sequential segments measuring approximately 0.5 cm, at about 1 cm from the cecum of the proximal colon of each animal were collected. These fragments were dissected and used for histopathological analysis and functional studies of the colon. 

### Histopathological analysis

The first segment extracted from the proximal colon of each animal in the experimental groups was fixed in 10% formaldehyde solution for 24 h and processed for inclusion in Paraplast Plus® (McCormick Scientific IL, USA). Then, 5 µm sections were obtained and stained with hematoxylin-eosin for analysis of the general morphology. To graduate the inflammatory process, the nests of amastigotes and degree of necrosis, a semi-quantitative scale from zero to three crosses was used^24^, being considered:

- inflammatory process:

“zero” (“-”) = absence of inflammation;

 “+” (<12.5% of fields) = mild inflammation;

“++” (25 to 50% of fields) = moderate inflammation;

 “+++” (50 to 100% of fields) = intense inflammation.

- amastigote nests:

“zero” (“-”): absence of amastigote nests;

“+”: only one amastigote nest;

“++”: two amastigote nests;

“+++”: three or more amastigote nests.

- necrosis:

“zero” (“-”): absence of necrosis;

“+”: only one focus of necrosis;

“++”: two foci of necrosis;

“+++”: three or more foci of necrosis.

### Colon functional characterization

The second segment collected from the colon was assembled by its ends in a longitudinal position, using metal hooks in vats containing 2 ml of Krebs-Henseleit nutrient, composed of 121.5 mM of NaCl; 4.7 mM KCl; 2.5 mM CaCl_2_; 1.2 mM KH_2_PO_4_; 1.2 mM MgSO_4_; 25 mM NaHCO_3_, and 5.6 mM glucose, at pH 7.4. It remained heated at 37 °C and was constantly infused with a carbogenic mixture in the proportion of 95% O_2_ and 5% CO_2_ ^25^. One of the metal hooks was connected to an adjustable fixed bracket, and the other was connected to an isometric force transducer (model ML221, ADInstruments, Australia). The changes in the tone of the preparations were recorded using a Powerlab® 8/30 data acquisition system (ADInstruments, Australia). During the 60 min preceding the beginning of the experimental protocol, the preparations were stabilized at rest under a tension of 1 g (10 mN). During this period and throughout the experiment, the nutrient solution was replaced every 15 min. Muscle reactivity was studied by obtaining concentration curves versus cumulative responses for noradrenaline (10^-^ ^10^ - 10^-^ ^4^ ^M^) and acetylcholine (10^-^ ^10^ - 10^-^ ^4^ ^M^), corresponding to relaxation and contraction, respectively. The increase in the concentration of potassium (K^+^) in the Krebs-Henseleit solution (depolarizing solution) was performed in parallel with a compensatory reduction in the concentration of sodium (Na^+^). To analyze and compare tissue responses between the different experimental groups, the values of maximum tissue response (MaxR) and the area under the curve (AUC) of the obtained graphics were calculated. Both parameters were calculated by non-linear regression using the Prism 4.0^®^ program (GraphPad Software, USA).

### Butyrylcholinesterase plasmatic activity

The plasma of each animal was thawed and the activity of the enzyme butyrylcholinesterase was determined in the DIMENSION® system, with absorbance readings at 600 and 700 nm and expressed in U/L. The experiments were carried out at the Biochemistry Laboratory of the Famema Hemocenter.

### Statistical analysis

The data obtained were presented as the mean ± standard deviation. The normality distribution was verified using the Shapiro-Wilk test. The homogeneity of the variances was analyzed using Levene’s test. A mixed ANOVA of repeated measures was carried out to analyze the effect of group, time, and interaction (group vs. time) on the conditions of dependent groups. The sphericity hypothesis was analyzed using Mauchly’s test, and when this was violated, the analyses were based on the Greenhouse-Geisser correction. Post-hoc comparisons were performed using the least significant difference (LSD) test. To analyze the effect of time and group in independent groups, two-way ANOVA was performed followed by the Bonferroni post-hoc comparison. However, when the hypothesis of homogeneity of variances was violated, the Games-Howell post-hoc test was used. The association between qualitative variables was assessed using Fisher's exact test. SPSS software (version 19.0) was used for all analyses, with a significance level of 5%.

## RESULTS

### Animal survival

During the experimental period, 13 animals died, of which two died at 30 dpi (INF=1, GAL=1), 6 at 60 dpi (CTRL=5, INF=1), and 5 at 90 dpi (CTRL=1, GAL=1, GAL/INF=1, INF=2). Samples from these animals were excluded from this study. 

### Parasitemia

**FIGURE 1:**
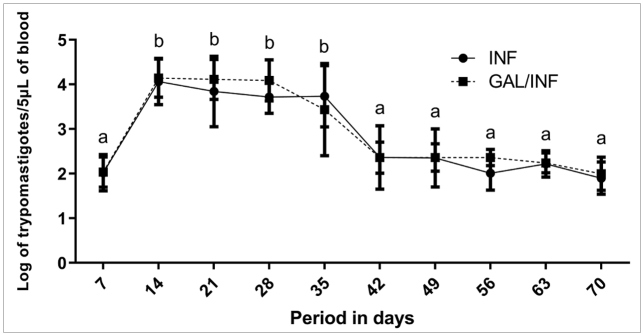
Parasitemic curve expressed by the logarithmic mean of the INF and GAL/INF groups during the evolution of the acute phase. **INF:** mice infected with *T. cruzi* QM2 strain and galantamine untreated; **GAL/INF:** mice infected with *T. cruzi* QM2 strain and galantamine-treated. Different letters correspond to significant differences between days for both groups, as determined by the post-hoc LSD test (p-value ≤ 0.05).

The logarithmic media of parasitemia measures obtained from five mice in each group (INF and GAL/INF) obtained at days 7, 14, 21, 28, 35, 42, 49, 56, 63, and 70 after infection, performed using the Anova Mista software, indicated a significant effect of time (days) on parasitemia, but without significant differences between groups. In the post-hoc analysis by the LSD test for the effect of time, both groups showed a significant increase at days 14, 21, 28, and 35 compared to day 7. Both groups also showed a significant reduction in parasitemia after day 35 (Figure 1).

### Histopathological analysis

**FIGURE 2:**
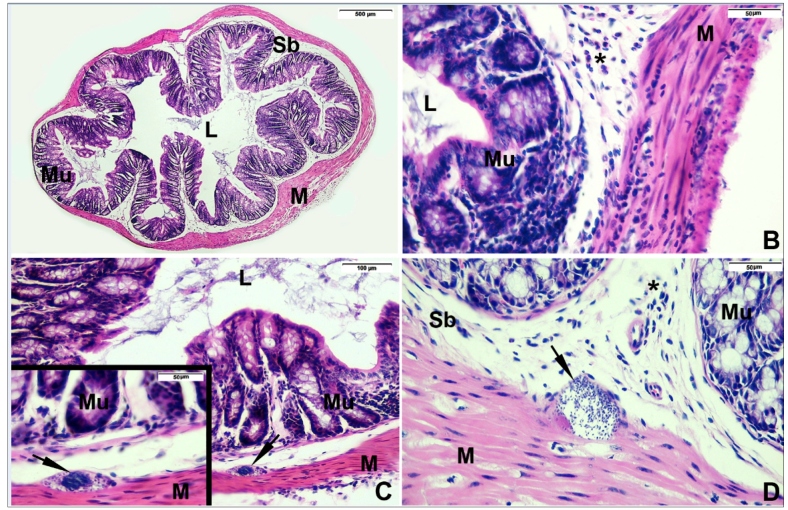
Photomicrographs of Swiss mice proximal colon histopathological characteristics.

Histopathological analysis of the proximal colon of mice infected with the *T. cruzi* QM2 strain is shown in Figure 2 and  Supplementary Material Table 1. The colon of animals from the INF group evaluated at 30 dpi showed the presence of a mild inflammatory infiltrate in all animals. In contrast, in 87.5% of the animals in the GAL/INF group, an inflammatory infiltrate was present; however, this was discrete in 75.0% and moderate in 12.5% (p = 0.999). The proximal colon of all animals evaluated at 60 and 90 dpi showed inflammatory infiltrates ranging from mild to moderate. Comparative analysis at day 60 showed a moderate inflammatory infiltrate in 75% of animals in the GAL/INF group and in 37.5% of the INF group (p = 0.315). At 90 dpi, 50% of the animals from the INF group and 55.6% of the GAL/INF group showed a moderate inflammatory process (p = 0.999). No significant difference was observed when comparing experimental times within each group individually, as well as when comparing one group in relation to the other. Amastigote nests were observed at 60 and 90 dpi, with no significant difference between them. The CTRL and GAL groups did not show significant inflammatory processes during the study period. No morphological signs of necrosis were observed in any of the animals studied. It is noteworthy that the reduction of animals in the histological study can be explained by tissue damage during the process of slide confection.

Staining: hematoxylin and eosin. Magnification: A = 40×; B, D, C-inset = 400×, C = 200×. (**A**) Animal treated with galantamine at day 90 after the start of the experiment (GAL-90), without inflammatory infiltrate or amastigote nest; (**B**) Animal infected with *T. cruzi* QM2 strain and treated with galantamine for 90 days (GAL/INF-90), exhibiting moderate inflammatory infiltrate (*); (**C**) Animal after 90 days of *T. cruzi* QM2 infection and untreated (INF-90), showing inflammatory mild infiltrate (*) and amastigote nest (arrow); (**C-inset**), detail of amastigote nest; (**D**) Animal after 60 days of infection with *T. cruzi* QM2 strain (INF-60), exhibiting mild inflammatory infiltrate (*) and amastigote nest (arrow). Abbreviations: **Sb:** submucosa; **L:** lumen; **M:** muscular layer; **Mu:** mucosa.

### Butyrylcholinesterase plasmatic activity

The mean values of the plasma activity of BuChE throughout the experimental period are shown in Table 1. A significant difference was observed in the values of the plasma activity of BuChE at day 30 after the beginning of the experiment when compared to the CTRL, GAL, and INF groups (p <0.001). Comparison of BuChE activity between the GAL/INF and INF groups revealed lower BuChE activity in the GAL/INF group, although the difference was not statistically significant, as well as for the CTRL and GAL groups. Lower BuChE activity was also observed at day 60 in the GAL group when compared with all other groups (p <0.014). Galantamine action was not observed among uninfected animals, and no significant difference was observed in the interaction between groups and time. 

**TABLE 1:** Mean and standard deviation comparison of BuChE plasmatic activity among experimental groups over time.

	Time	CTRL	GAL	INF	GAL/INF
BuChE	30 days	5235 ± 679ᶲ	5301 ± 1093ᶲ	7807 ± 1224	6058 ± 1547
	60 days	4873 ± 836	4200 ±1025ᶲø	6647 ± 662	6841 ± 2510
	90 days	4614 ± 734	4445 ± 629	5946 ± 1148†	5520 ± 598

Data are expressed as the mean ± standard deviation. Statistically significant difference at day 30 (†) after the beginning of the experiment, and over time in INF (ᶲ) and GAL/INF (ø) groups, according to the post-hoc test (p≤ 0.05).

### Colon functional evaluation

**FIGURE 3:**
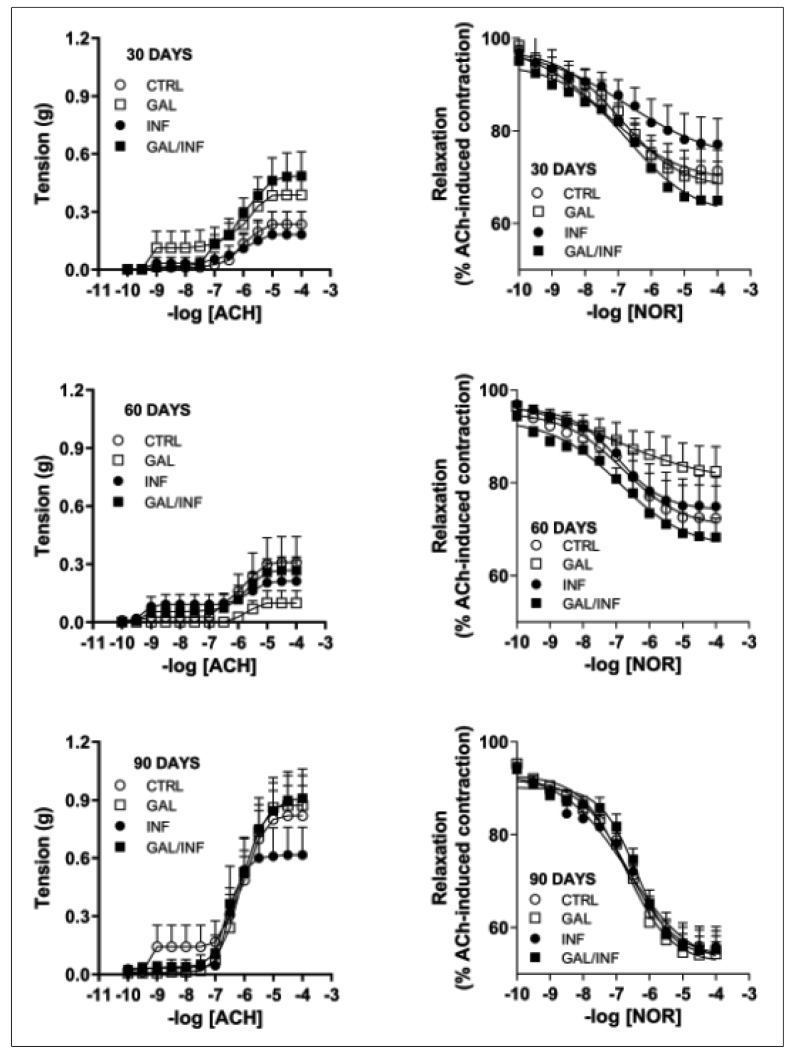
Colonic muscle average tension obtained after pipetting ACh and NOR (10^-10^ to 10^-4^ M) treatment among groups over time.

The colonic muscle reactivity of the mice from the experimental groups at 30, 60 and 90 dpi is shown in Figure 3 and Table 2 (AUC). The MaxR values of reactivity are available in  Supplementary Material Tables 2 **and** 3. Mice colons from the INF group at 30 dpi responded to the action of stimulating drugs similar to the CTRL group. At day 30 after the experimental procedure, the GAL and GAL/INF groups showed the greatest effect of galantamine on both the contraction and relaxation curves. The INF and GAL/INF animal groups showed a lower response to ACh and NOR stimuli at day 60 after the beginning of the experiment. An increase in colonic muscle contraction and relaxation capacity was observed in the *T. cruzi* QM2 infected groups at day 90.

**TABLE 2:** Comparison of the mean and standard deviation of the AUC values of ACh and NOR treatment among the experimental groups over time.

		Experimental groups			
	Time (days)	CTRL	GAL	INF	GAL/INF
**ACh**	30	0.52 ± 0.49	1.15 ± 1.31	0.5 ± 0.71	1.13 ± 0.91
	60	0.71 ± 0.81	0.18 ± 0.3	0.69 ± 1.04	0.67 ± 0.57
	90	2.13 ± 1.6 †	1.90 ± 1.14 ‡	1.61 ± 1.34	2.07 ± 1.73 ‡
**NOR**	30	492.2 ± 87.4	496.2 ± 36.4	518.8 ± 53.5	476 ± 64.5
	60	501.1 ± 69.6	533.8 ± 55.9	511.5 ± 43	480.9 ± 43.2
	90	447.5 ± 32.7	445.3 ± 42.9 †‡	445.8 ± 43.2 †‡	452.4 ± 22.9

Values are expressed as the mean ± standard deviation. **AUC:** area under the curve. Statistically significant difference at day 30 (†) and day 60 (‡) among experimental groups according to post-hoc test (p ≤ 0.05).

## DISCUSSION

The digestive manifestations of Chagas disease, known as megaesophagus and megacolon, are consequences of the inflammatory response deflagrated at the onset of the infection. *T. cruzi* epitopes are recognized by Toll- and NOD-like receptors, found in macrophages and dendritic cells. This initiates an innate immune response^26^ ^-^ ^27^ and stimulates the synthesis of inflammatory mediators and the activation and differentiation of Th1 and Th2 lymphocytes, depending on the relationship between the host and the parasite strain^28^ ^-^ ^29^, in an attempt to contain parasitic multiplication. Consequently, there is an exacerbated production of cytokines that stimulate inflammatory and anti-inflammatory signals, including the efferent anti-inflammatory signal known as the anti-inflammatory cholinergic pathway^13^ ^,^ ^14^ ^,^ ^30^. The interaction between the nervous and immune systems occurs through the afferent pathways of the vagus nerve, culminating in the activation of β2 adrenergic receptors in specialized T lymphocytes of the spleen, increasing the synthesis of ACh^31^, which leads to inflammatory cytokine suppression by several cells that have receptors for ACh, the main one being macrophages^13^ ^,^ ^32^ ^,^ ^33^, without observing suppression of anti-inflammatory cytokines such as IL -10^34^. 

In this context, it is possible to infer that the use of anticholinesterase drugs such as galantamine would contribute to increased parasitic multiplication by reducing the degradation of acetylcholine. Consequently, there would be a decrease in the synthesis of pro-inflammatory cytokines, which are synthesized to contain *T. cruzi* multiplication, thus aggravating the evolution of the initial and intermediate stages of Chagas disease. According to other research carried out by our group^35^, this inflammatory process triggered at the beginning of the infection is fundamental for the control of *T. cruzi* multiplication. However, the results of this study did not show statistically significant differences in parasitemia between the INF and GAL/INF groups, as well as in the histopathological analysis, where no statistical differences were observed in the inflammatory process and colonic involvement by amastigote nests during disease progression. Although a greater number of animals displaying a moderate degree of inflammatory infiltrates were observed in the GAL/INF group at 60 dpi, this increase was not maintained at the beginning of the chronic phase, making it impossible to demonstrate our premise. The dosage of galantamine administered to the animals in this study may have interfered with these results, as well as the mechanism of action of this drug, which has a high selectivity for AChE when compared to BuChE^36^ ^-^ ^38^. Thus, due to the low inhibitory capacity of BuChE, ACh hydrolysis would continue to occur normally, preventing its binding to the α7AChR macrophage receptor, which is responsible for inhibiting IKB and phosphorylation of JAK2, with consequent dimerization of STAT3 - pathways that act simultaneously, hence inhibiting nuclear factor κB (NF-κB) transcription, resulting in reduced inflammatory cytokine synthesis^30^ ^,^ ^39^ ^,^ ^40^. 

Studies by Zanella et al.^29^ and Campello et al.^41^ demonstrated variations in BuChE concentrations in Chagas disease, both in the acute and chronic phases, indicating its role as an inflammatory marker^25^ ^,^ ^42^. These results corroborated the values of BuChE activity observed in the INF group, which were significantly higher than those observed in the CTRL and GAL groups. The absence of statistically significant differences in plasma BuChE activity between the GAL/INF group and the other groups studied reinforces the mechanism of action of galantamine on AChE, as previously reported. According to Upadhyay et al.,^43^ galantamine presents a selectivity that is 10 times more than AChE in relation to BuChE. 

Despite the decrease in BuChE activity at the end of the acute phase (60 days after *T. cruzi* infection) and at the beginning of the chronic phase (90 days after *T. cruzi* infection) in the INF and GAL/INF groups, probably due to the reduction in parasitemia, there was also a tendency toward a reduction in BuChE activity over the experimental period in the control groups. Considering that there may be a relationship between BuChE and AChE activity, it is possible to infer that the enzymatic degradation of ACh is greater in the cholinergic synapses located in the colons of younger animals. This hypothesis corroborates the findings of Phillips et al.^44^ and Thrasivoulou et al.,^45^ who found deterioration of the submucosal plexus and loss of myenteric neurons, respectively, in the colon of rats from adulthood. In this context, Carlucci et al.^46^ showed that the reduction in AChE activity was related to the degree of impairment of cholinergic innervation in the stomachs of chronic chagasic patients. This probable neuronal loss that occurs physiologically with aging may explain the ACh responses in terms of MaxR and ASC, which were greater in the colon preparations of animals studied at 90 days post infection in the four experimental groups, although not significant. Regardless of the BuChE values being significantly different in this period between the non-infected groups (CTRL and GAL) and the INF group, we observed no difference in responses to ACh, suggesting that a possible relationship between BuChE and AChE activity should not be linear. The absence of statistically significant differences in Rmax or ASC in the concentration-response curves for ACh between the different experimental groups studied at 30, 60, and 90 days after infection suggests that the inflammatory process caused by infection with *T. cruzi* was not able to alter the colon responses of these animals to ACh. According to Carlucci et al.^46^, functional changes in the digestive system depend on the intensity of denervation. In addition, in this study, it was not possible to demonstrate the action of galantamine in the responses to ACh in uninfected animals or in animals submitted to infection. 

The results of this study showed that the colon responses to NOR were higher in the animals studied at 90 days after infection, similar to ACh. It should be noted, however, that responses to NOR were obtained in preparations previously contracted with ACh, expressed as a percentage of the contraction induced by the agonist. In this way, we believe that the greater responses to NOR were a consequence of the fact that ACh also promoted more vigorous contractions in this preparation. Similarly, relaxing responses to NOR were also greater in the presence of a stronger pre-contraction. Therefore, as with ACh, infection and treatment with galantamine did not change the responses of these preparations to NOR.

The results obtained in this study did not corroborate the anti-inflammatory effect of galantamine in experimental Chagas disease, as demonstrated in other previously mentioned experimental models. However, this hypothesis cannot be completely ruled out, as the study was limited by the non-measurement of the plasmatic activity of AChE and concentration of pro-inflammatory cytokines, as well as by the restricted experimental time, which did not allow a more extensive assessment of the chronic phase of the infection. Future studies should be carried out using different concentrations of galantamine and other *T. cruzi* strains. 
