# Supplementary figures and images for: Influence of galantamine in the inflammatory process and tissular lesions caused by Trypanosoma cruzi QM2 strain
Source: Rev Soc Bras Med Trop. 2021 Nov 12;54:e0201-2021. doi: 10.1590/0037-8682-0201-2021 (PMC8582970; doi:10.1590/0037-8682-0201-2021)

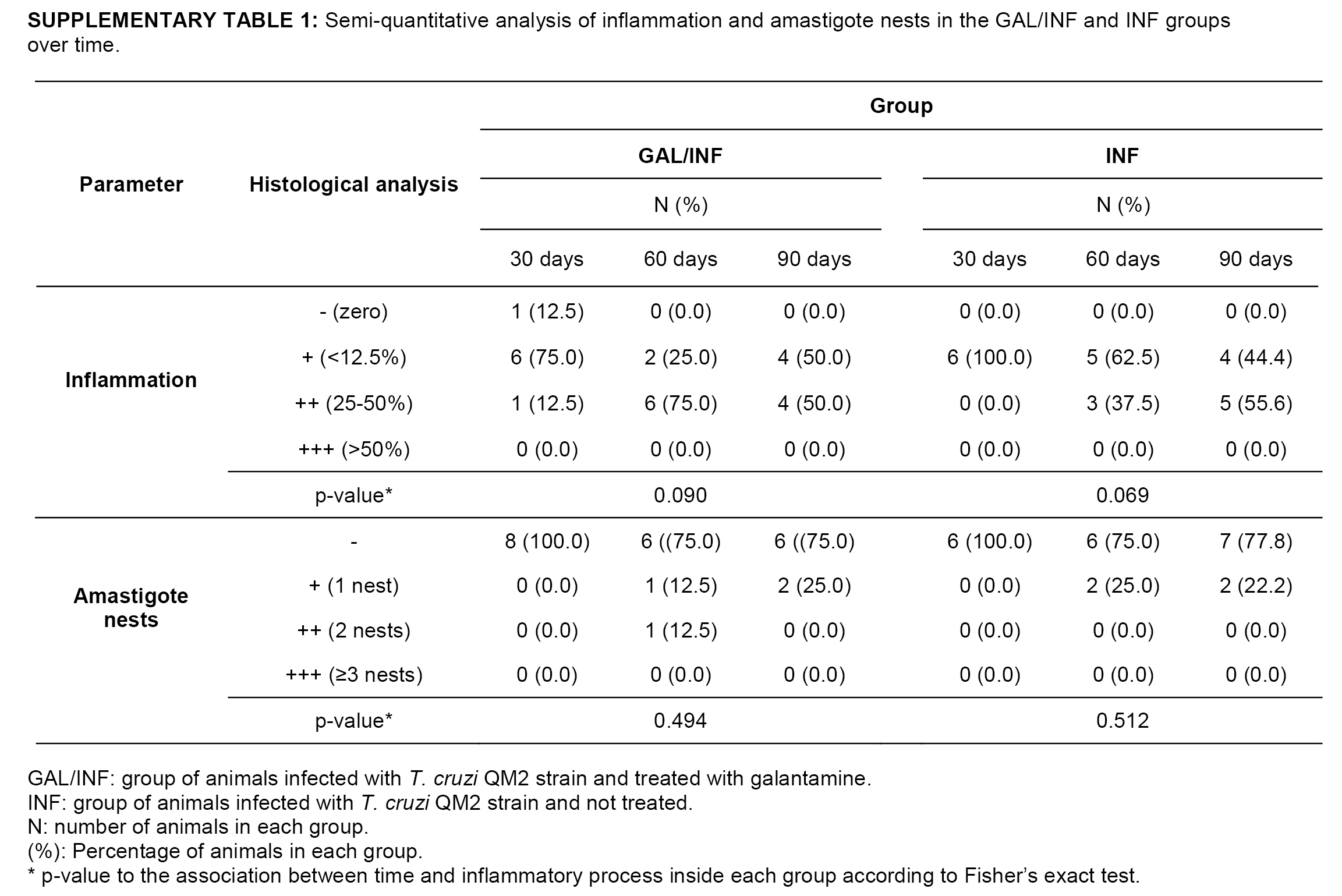

Supplement: Supplementary file 1 [file 1678-9849-rsbmt-54-e0201-2021-supp1.jpg]

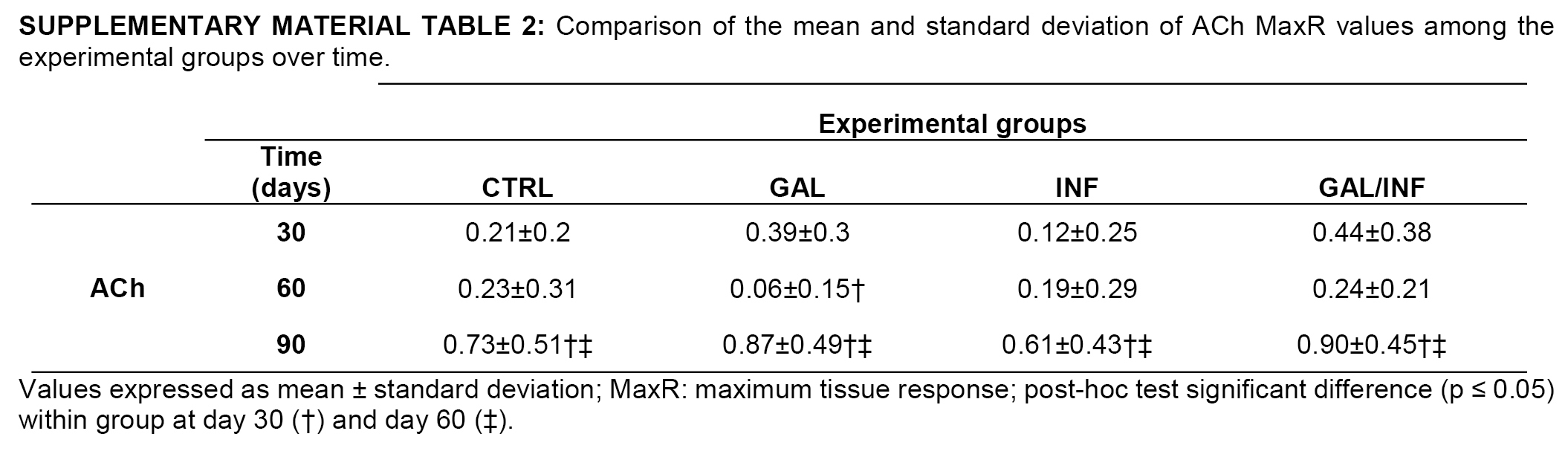

Supplement: Supplementary file 2 [file 1678-9849-rsbmt-54-e0201-2021-supp2.jpg]

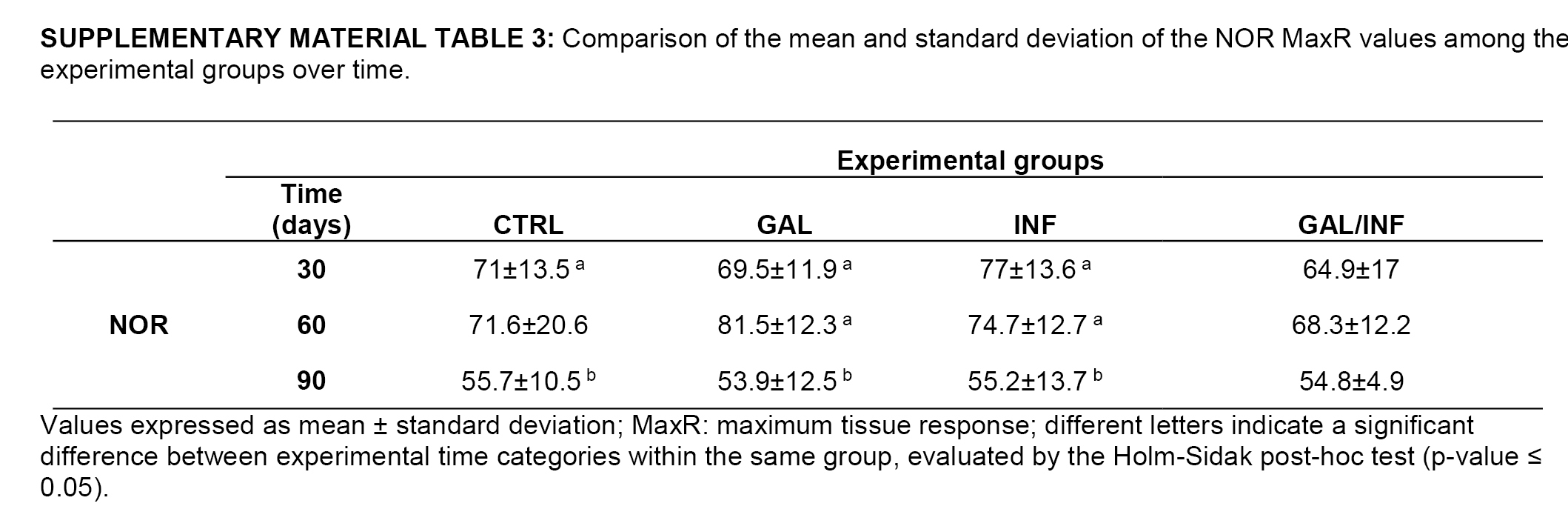

Supplement: Supplementary file 3 [file 1678-9849-rsbmt-54-e0201-2021-supp3.jpg]
